# Molecular Inconsistencies in a Fragile X Male with Early Onset Ataxia

**DOI:** 10.3390/genes7090068

**Published:** 2016-09-21

**Authors:** Yun Tae Hwang, Tracy Dudding, Solange Mabel Aliaga, Marta Arpone, David Francis, Xin Li, Howard Robert Slater, Carolyn Rogers, Lesley Bretherton, Desirée du Sart, Robert Heard, David Eugeny Godler

**Affiliations:** 1 Department of Neurology, Gosford Hospital, Gosford 2250, Australia; y.hwang@ucl.ac.uk; 2 Institute of Neurology, University College London, London WC1N 3BG, UK; 3 Genetics of Learning Disability Service, Hunger Genetics, Waratah 2298, Australia; Tracy.Dudding@hnehealth.nsw.gov.au (T.D.); Carolyn.Rogers@hnehealth.nsw.gov.au (C.R.); 4 Grow Up Well Priority Research Centre, University of Newcastle, Waratah 2308, Australia; 5 Cyto-molecular Diagnostic Research Laboratory, Victorian Clinical Genetics Services and Murdoch Childrens Research Institute, Royal Children’s Hospital, Melbourne 3052, Australia; solange.aliagavera@mcri.edu.au (S.M.A.); marta.arpone@mcri.edu.au (M.A.); david.francis@vcgs.org.au (D.F.); xin.li@mcri.edu.au (X.L.); howard.slater@vcgs.org.au (H.R.S.); 6 Faculty of Medicine, Dentistry and Health Sciences, University of Melbourne, Melbourne 3010, Australia; 7 Molecular and Cytogenetics Laboratory, INTA University of Chile, Santiago 7830490, Chile; 8 Psychology Service, Royal Children’s Hospital, Melbourne 3052, Australia; Lesley.Bretherton@rch.org.au; 9 Melbourne School of Psychological Sciences, University of Melbourne, Melbourne 3052, Australia; 10 Child Neuropsychology, Murdoch Childrens Research Institute, Royal Children’s Hospital, Melbourne 3052, Australia; 11 Molecular Genetics Laboratory, Victorian Clinical Genetics Services and Murdoch Childrens Research Institute, Royal children’s Hospital, Melbourne 3052, Australia; dusart.desiree@mcri.edu.au; 12 Faculty of Medicine, University of Newcastle, Newcastle 2303, Australia; morkeu@gmail.com; 13 Westmead Millennium Institute, University of Sydney, Westmead 2145, Australia

**Keywords:** fragile X syndrome, *FMR1*, FXTAS, ataxia, tremor, methylation, mosaicism, AmplideX, Southern blot, RNA toxicity

## Abstract

Mosaicism for *FMR1* premutation (PM: 55–199 CGG)/full mutation (FM: >200 CGG) alleles or the presence of unmethylated FM (UFM) have been associated with a less severe fragile X syndrome (FXS) phenotype and fragile X associated tremor/ataxia syndrome (FXTAS)—a late onset neurodegenerative disorder. We describe a 38 year old male carrying a 100% methylated FM detected with Southern blot (SB), which is consistent with complete silencing of *FMR1* and a diagnosis of fragile X syndrome. However, his formal cognitive scores were not at the most severe end of the FXS phenotype and he displayed tremor and ataxic gait. With the association of UFM with FXTAS, we speculated that his ataxia might be related to an undetected proportion of UFM alleles. Such UFM alleles were confirmed by more sensitive PCR based methylation testing showing FM methylation between 60% and 70% in blood, buccal, and saliva samples and real-time PCR analysis showing incomplete silencing of *FMR1*. While he did not meet diagnostic criteria for FXTAS based on MRI findings, the underlying cause of his ataxia may be related to UFM alleles not detected by SB, and follow-up clinical and molecular assessment are justified if his symptoms worsen.

## 1. Introduction

CGG expansions of more than 200 CGG repeats within the 5′untranslated end of *FMR1* are termed full mutations (FM) and are associated with epigenetic silencing of the *FMR1* gene [1]. Absence of *FMR1* transcription usually results from abnormal methylation of the *FMR1* promoter [2] and is associated with loss of *FMR1* protein (FMRP). Absence of FMRP, which is a protein essential for normal neurodevelopment, is the primary cause of the fragile X syndrome (FXS) [3]. FXS is the most common monogenic cause of autism and intellectual disability [4] with a prevalence of 1 in 4000 males and 1 in 8000 females in the general population [5].

The more common CGG triplet expansions of 55 to 199 CGG repeats are termed premutations (PM) and are found in approximately 1 in 450 males and 1 in 150 females in the general population [6]. In contrast to FM, PM alleles have a completely unmethylated *FMR1* promoter similar to normal size alleles with less than 44 repeats [7], but are associated with elevated *FMR1* mRNA levels and paradoxically impaired translation and decreased FMRP levels [8]. Increased *FMR1* transcription usually leads to gain-of-function toxicity resulting in aggregation of proteins by the excessive mRNA, formation of ubiquitin-positive inclusion bodies in the brain and other tissues, mitochondrial dysfunction, and excessive cell death [9,10,11]. This molecular cascade has been postulated to be a major contributing factor in the development of PM-specific disorders including fragile X tremor and ataxia syndrome (FXTAS)—a neurodegenerative disorder affecting up to 45% PM males and 17% PM females over the age of 50 [12]. Other molecular contributors to PM specific phenotypes have been suggested to be decreased FMRP levels [8], over-expression of long non-coding RNA of *ASFMR1/FMR4* [11], *FMR5*, and *FMR6* [13], and repeat associated non-ATG translation [14].

Although FXS and FXTAS are regarded as distinct disorders, several lines of evidence now suggest that they can be comorbid [15]. Specifically, diagnosis of probable FXTAS has been reported in three FM male cases with completely unmethylated FM alleles in blood [16,17,18]. While significant increases in *FMR1* mRNA were reported in blood from all three cases, CGG size testing in other peripheral tissues identified PM/FM mosaicism for one of these [18]. More recently, we have described tissue mosaicism for FM methylation and a microdeletion proximal to the CGG repeat in a 33 year old male with clinical and MRI findings consistent with FXTAS, which is the youngest case reported to date of probable FXTAS [19].

In this study, we describe a 38 year old FXS male with a fully methylated *FMR1* promoter in blood, detected using Southern blot analysis of the *NruI* restriction site within the *FMR1* CpG island. This approach is the gold standard FXS diagnostic test where full methylation is understood to indicate complete silencing of *FMR1* [20]. However, this result was at odds with methylation analysis of other regions within the *FMR1* promoter and *FMR1* expression that was reduced, but not silenced. While this individual did not meet the diagnostic criteria for FXTAS, he did show early onset ataxia in his 30s.

## 2. Experimental Section

### 2.1. Ethics Approval

The follow-up clinical and molecular assessments consequent to assessment of his tremor and ataxia symptoms in the neurology clinic were conducted as part of the FREE FX study, approved by The Royal Children’s Hospital Research and Ethics Committee.

### 2.2. Sample Processing

CGG triplet repeat sizing and methylation testing were performed on 15 mL of peripheral blood collected in EDTA tubes, saliva samples collected using the Oragene^®^ DNA Self-Collection Kit (DNA Genotek, Ottawa, ONT, Canada), and buccal samples collected using the Master Amp Buccal Swab Brush kit (Epicentre Technologies, Madison, WI, USA). DNA was isolated from these sample types using the NucleoSpin^®^ Tissue genomic DNA extraction kit (Machery-Nagel, Duren, Germany). Five mL of blood was used for peripheral blood mononuclear cell (PBMC) isolation using Ficoll gradient separation [10]. RNA was extracted from the isolated PBMCs using RNeasy kit (Qiagen Global, Waldbronn, Germany) as per the manufacturer’s instructions.

### 2.3. Molecular Analyses

Initial CGG repeat sizing in all samples was performed using polymerase chain reaction (PCR) as previously described [21]. Methylation-sensitive Southern blot was used to confirm presence of a FM allele and to perform methylation analysis of the *NruI* restriction site located within the CpG island 5′ of the CGG expansion, which is the “gold standard” recommended in FXS diagnostics [20]. Methylation controls included in the Southern blot test were DNA samples from a male and female with normal CGG size alleles and male and female FM carriers as described in a previous publication [2]. Five μg of DNA were digested with the methylation-sensitive enzyme combination of *NruI*/*HindIII*. The DNA probe *Pfxa3a* was radio-labelled with P^32^ (Random primer DNA labelling kit, TAKARABIO, Otsu, Japan) and used to detect *FMR1* allele-specific restriction fragments by autoradiography as previously described [22]. The AmplideX™ *FMR1* PCR Kit (Asuragen, Austin, TX, USA) was used to confirm CGG triplet repeat sizing and to perform methylation analysis of two *HpaII* sites: 5′ and 3′ of the CGG expansion using blood DNA.

**Figure 1:**
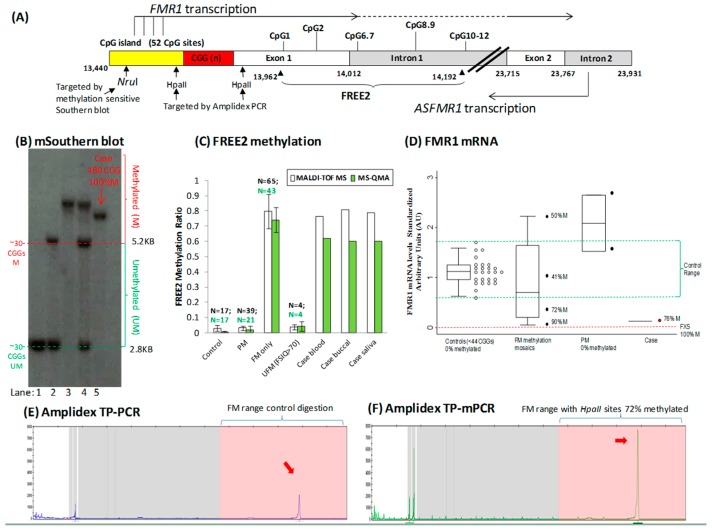
Molecular results. (**A**) Organization of the *FMR1* 5′ region including the CGG expansion (sequence numbering from GenBank L29074 L38501) in relation to *FMR1* and *ASFMR1* transcription start sites (the broken lines indicate spliced out regions), fragile X related epigenetic element 2 (FREE2), the *FMR1* CpG island and methylation sensitive restriction site *NruI* analysed using routine fragile X Southern blot testing, two *HpaII* sites targeted by AmplideX methylation PCR. The FREE2 region is located downstream of the CGG expansion, at the exon 1/intron1 boundary and includes 12 CpG sites. (**B**) Methylation sensitive Southern blot analysis of the *NruI* restriction site within *FMR1* CpG island in blood. The DNA sample in question from the fragile X syndrome (FXS) male suspected of fragile X associated tremor/ataxia syndrome (FXTAS) is in lane 5. Comparator DNA sized using standard CGG PCR^1^ from: (1) a typical male and a female (CGG < 44) are in lanes 1 and 2; (2) full mutation (FM) male with a 100% methylated 613 CGG allele is in lane 3; (3) FM female with a 100% methylated 580 CGG allele is in lane 4. (**C**) Mean methylation output ratio of CpG sites located within the FREE2 region. Assessed using MALDI-TOF MS and MS-QMA^2^. Note: the error bars for reference ranges represent one standard deviation from the mean FREE2 methylation in blood of male controls (CGG < 44); Premutation (PM) males (56–170 CGGs); FM males with typical FXS (213–2000 CGGs), and four atypical “high functioning” unmethlyated full mutation (UFM) males by Southern blot (CGG 200–637 CGG). The reference samples were co-run with DNA from the FXS male case suspected of FXTAS from blood, buccal, and saliva samples in question. (**D**) *FMR1* mRNA assessed using real-time PCR relative standard curve method. AmplideX PCR targeting methylation of two *HpaII* sites. (**E**) Hexachloto-Fluorescein (HEX) and (**F**) Fluorescein-Amidite (FAM) channels from capillary electrophoresis of the DNA from the blood of the male case in question. Note: red arrows indicate presence of positive FM alleles with methylated *HpaII* sites and the control digestion (*HpaII* methylation independent).

The Sequenom MALDI-TOF MS EpiTYPER system (Sequenom, USA) and Methylation Specific Quantitative Melt Analysis (MS-QMA) were used to analyse mean methylation of the fragile X related epigenetic element 2 (FREE2, located 3′ of the CGG repeat) in blood, buccal, and saliva samples as previously described [23]. Specifically, duplicate bisulfite conversions were performed for each DNA sample, with each conversion analysed twice using the EpiTYPER system and MS-QMA. The average of the four methylation output measurements per sample was used as a summary measure of mean FREE2 methylation presented in Figure 1.

*FMR1* mRNA analysis was performed using the relative standard curve real-time PCR method, where the mean of *FMR1*-5′ and *FMR1*-3′ assay outputs was normalized to average expression of three internal control genes (*GUS*, *EIF4A2*, and *SDHA*), as previously described [11]. For each sample, RNA was reverse transcribed in two separate cDNA reactions and then each was analysed in two separate real time-PCR reactions. The mean of the four arbitrary unit outputs was used as a summary measure for *FMR1* mRNA expression for each sample.

### 2.4. Ruling Out Other Causes of Ataxia

PCR and fragment analyses were used to examine repeat regions associated Friedreich’s ataxia (FA) and spinocerebellar ataxias (SCA) as part of the molecular diagnostics follow-up, using standard diagnostic protocols as previously described [24,25].

## 3. Results and Discussion

### 3.1. Clinical Features and Medical History

A 38 year old intellectually disabled male with a previous diagnosis of fragile X syndrome presented to a neurology clinic with a history of intermittent tremor and increasing unsteadiness over the previous several months. His medical history included type 2 diabetes mellitus and hyperthyroidism due to Grave’s disease. His medications included metformin, gliclazide, insulin, telmisartan, and rabeprazole. His living arrangements were semi-supervised in a flat downstairs from his parents. He has no family history of other movement disorders, including essential tremor; he has only consumed small amounts of alcohol in social settings and there was no history of substance abuse.

**Figure 2:**
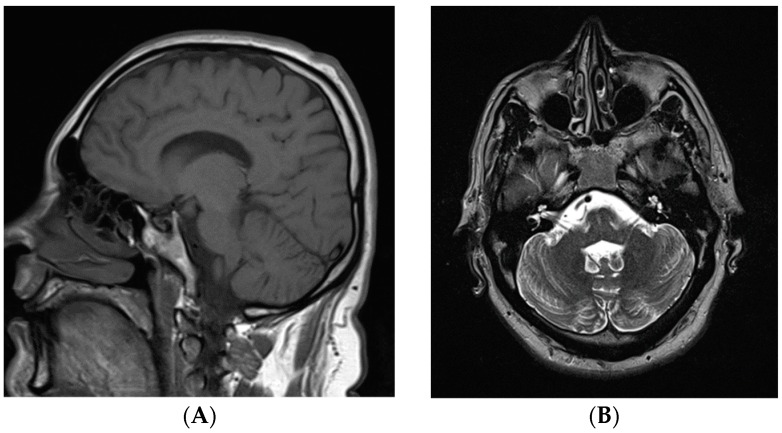
Magnetic resonance imaging of the brain. (**A**) T1 weighted sagittal view; and (**B**) T2 weighted axial view. Both images demonstrate the normal appearance of the cerebellum.

His physical examination demonstrated a high frequency of up-down action tremor without any resting tremor, truncal tremor, titubation, or extrapyramidal signs. Although he had no cerebellar signs in his limbs or nystagmus, he demonstrated a slightly wide-based gait and was unable to complete tandem gait suggesting a mild degree of cerebellar dysfunction. No other involuntary movements or pyramidal signs or apraxia were present. His brain magnetic resonance imaging revealed mild cerebellar atrophy (Figure 2).

Formal assessments of cognitive and behavioural impairments previously associated with the FXS phenotype were performed at 38 years of age, six months after the initial visit. While he was cognitively impaired with full scale IQ (FSIQ) of 50 [26], the score was above the mean FSIQ of 41.2, previously reported in a sample of 51 males with fully methylated FM alleles [27]. However, his overall Autism Diagnostic Observational Schedule-2 (ADOS-2) calibrated severity score (CSS) of 9 [28,29] indicated that he has significant autism spectrum symptomology. His Aberrant Behaviour Checklist Community (ABC-C) scores on Stereotypy and Inappropriate Speech were consistent with individuals with FXS [29] and with the repetitive speech and behaviours observed during the ADOS-2 assessment. However, his ABC-C score on three subscales, (Irritability, Lethargy, and Hyperactivity) were much lower than the mean scores obtained by adult males with FXS [30]. His scores on the Adult Behaviour Checklist (ABCL) were in the normal range for the Externalizing Scale, borderline range for the Internalizing Scale with Somatic Complaints score in the clinically significant range [31].

Blood biochemistry investigations, including thyroid function tests, vitamin B12, folate, autoimmune, and paraneoplastic antibody panel, were all within normal limits. Despite a history of suboptimally controlled diabetes, there was no evidence of sensory or motor neuropathy on the nerve conduction studies, ruling out other possible causes of tremor and ataxia. While PCR based testing for other causes of ataxia did not support Friedrich’s ataxia or spinocerebellar ataxias type 1, 2, 3, 6, and 7, it is possible that the FXS patient has another neurological disorder that would explain the early onset ataxia.

### 3.2. Genetic Testing

The original diagnostic investigation for FXS at 14 years of age, based on morphological features and cytogenetic testing of cells expressing the Xq27.3 fragile site, was negative. At 38 years of age, he was again referred for FXS testing after his nephew was diagnosed with this syndrome. The follow-up testing using Southern blot revealed a FM expansion but did not use methylation-sensitive enzyme/s. Methylation sensitive Southern blot analysis performed that same year identified a FM allele of 480 CGG repeats that had a 100% methylated *NruI* site within the *FMR1* CpG island (Figure 1B). This, at first, appeared to rule out the presence of PM or unmethylated FM alleles. However, methylation of the FREE2 region in blood, saliva, and buccal cells using MALDI-TOF MS and MS-QMA revealed partial methylation of the *FMR1* promoter (60%–70%). Presence of partially methylated FM alleles was further confirmed by (i) AmplideX TP-mPCR through analysis of *HpaII* site methylation on either side of the CGG repeat (72% methylation) (Figure 1F) and (ii) Analysis of *FMR1* mRNA levels in PBMCs revealing presence of transcription at 11% of the control group median levels. This suggested that *FMR1* mRNA was not completely silenced in some FM alleles that could explain (i) RNA toxicity at least in a minority of cells and (ii) observed symptoms of ataxia. This is consistent with another 33 year old male case where tissue mosaicism for FM methylation, similarly, did not show *FMR1* mRNA levels above the normal range, however, he had clinical and MRI findings consistent with FXTAS [19].

## 4. Conclusions

This FXS patient demonstrated early onset ataxia without MRI changes beyond mild cerebellar atrophy. While his Southern blot results showed a FM with complete methylation of the *FMR1* promoter, from which it could be inferred that the gene was completely silenced in all of his cells, this was inconsistent with the following results: (i) reduced but not silenced *FMR1* transcription; (ii) blood biochemistry results ruling out other causes of ataxia; (iii) his cognitive impairment being not as severe as previously reported in most fully methylated FM males. All of this suggested that in this FXS male, FM alleles in a proportion of cells had unmethylated FM alleles and thus were able to express potentially toxic *FMR1* mRNA. This was supported by FREE2 methylation testing in blood, buccal, and saliva, AmplideX methylation testing, and *FMR1* mRNA analysis in blood. Two potential explanations for the discordance in the methylation results obtained between Southern blot and the other techniques are: (i) the analytical sensitivity of Southern blot methylation analysis is 20% [23], meaning alleles that are 80% methylated will appear as if they are 100% methylated; (ii) the *NruI* restriction site methylation is different to methylation of the *HpaII* restriction sites targeted by AmplideX and the FREE2 region targeted by MALDI-TOF MS and MS-QMA. Together this evidence supports the conclusion that *FMR1* mRNA is expressed from expanded FM alleles that may be toxic, even in individuals who appear to have a fully methylated FM by the “gold standard” methylation-sensitive Southern blot test. Furthermore, co-morbidity of FXS and ataxia may be more common than previously realised as ataxia has been noted in some older FXS FM patients identified using methylation-sensitive Southern blot in previously described cohorts [32]. We, therefore, propose that if an FXS patient develops ataxia, additional testing be performed including DNA methylation analysis of multiple sites within the *FMR1* promoter (especially the FREE2 region) and in multiple tissues, along with testing for *FMR1* expression analysis if RNA is available. This additional diagnostic information may better explain the clinical phenotype and provide some assurance to the patients and their family that this is another manifestation of the *FMR1* PM or unmethylated FM alleles, rather than a new, unexplained aetiology. Whether the underlying cause of this patient’s ataxia is the same as for FXTAS is unknown, but follow-up study is justified to consider if his symptoms worsen.
