# Erratum: Hwang Y.T. et al. Molecular Inconsistencies in a Fragile X Male with Early Onset Ataxia. *Genes* 2016, *7*, 68

**DOI:** 10.3390/genes8020047

**Published:** 2017-01-24

**Authors:** Yun Tae Hwang, Tracy Dudding, Solange Mabel Aliaga, Marta Arpone, David Francis, Xin Li, Howard Robert Slater, Carolyn Rogers, Lesley Bretherton, Desirée du Sart, Robert Heard, David Eugeny Godler

**Affiliations:** 1 Department of Neurology, Gosford Hospital, Gosford 2250, Australia; y.hwang@ucl.ac.uk; 2 Institute of Neurology, University College London, London WC1N 3BG, UK; 3 Genetics of Learning Disability Service, Hunger Genetics, Waratah 2298, Australia; Tracy.Dudding@hnehealth.nsw.gov.au (T.D.); Carolyn.Rogers@hnehealth.nsw.gov.au (C.R.); 4 Grow Up Well Priority Research Centre, University of Newcastle, Waratah 2308, Australia; 5 Cyto-molecular Diagnostic Research Laboratory, Victorian Clinical Genetics Services and Murdoch Childrens Research Institute, Royal Children’s Hospital, Melbourne 3052, Australia; solange.aliagavera@mcri.edu.au (S.M.A.); marta.arpone@mcri.edu.au (M.A.); david.francis@vcgs.org.au (D.F.); xin.li@mcri.edu.au (X.L.); howard.slater@vcgs.org.au (H.R.S.); 6 Faculty of Medicine, Dentistry and Health Sciences, University of Melbourne, Melbourne 3010, Australia; 7 Molecular and Cytogenetics Laboratory, INTA University of Chile, Santiago 7830490, Chile; 8 Psychology Service, Royal Children’s Hospital, Melbourne 3052, Australia; Lesley.Bretherton@rch.org.au; 9 Melbourne School of Psychological Sciences, University of Melbourne, Melbourne 3052, Australia; 10 Child Neuropsychology, Murdoch Childrens Research Institute, Royal Children’s Hospital, Melbourne 3052, Australia; 11 Molecular Genetics Laboratory, Victorian Clinical Genetics Services and Murdoch Childrens Research Institute, Royal children’s Hospital, Melbourne 3052, Australia; dusart.desiree@mcri.edu.au; 12 Faculty of Medicine, University of Newcastle, Newcastle 2303, Australia; morkeu@gmail.com; 13 Westmead Millennium Institute, University of Sydney, Westmead 2145, Australia

The authors wish to make the following correction to their paper [1]. The authors’ affiliations are wrong and should be replaced with:

Yun Tae Hwang ^1,2^, Tracy Dudding ^3,4^, Solange Mabel Aliaga ^5,6,7^, Marta Arpone ^5,6,10^, David Francis ^5^, Xin Li ^5^, Howard Robert Slater ^5,6^, Carolyn Rogers ^3^, Lesley Bretherton ^8,9,10^, Desirée du Sart ^11^, Robert Heard ^12,13,†^ and David Eugeny Godler ^5,^*^,†^

^1^  Department of Neurology, Gosford Hospital, Gosford 2250, Australia; y.hwang@ucl.ac.uk
^2^  Institute of Neurology, University College London, London WC1N 3BG, UK
^3^  Genetics of Learning Disability Service, Hunter Genetics, Waratah 2298, Australia; Tracy.Dudding@hnehealth.nsw.gov.au (T.D.); Carolyn.Rogers@hnehealth.nsw.gov.au (C.R.)
^4^  Grow Up Well Priority Research Centre, University of Newcastle, Waratah 2308, Australia
^5^  Cyto-molecular Diagnostic Research Laboratory, Victorian Clinical Genetics Services and Murdoch Childrens Research Institute, Royal Children’s Hospital, Melbourne 3052, Australia; solange.aliagavera@mcri.edu.au (S.M.A.); marta.arpone@mcri.edu.au (M.A.); david.francis@vcgs.org.au (D.F.); xin.li@mcri.edu.au (X.L.); howard.slater@vcgs.org.au (H.R.S.)
^6^  Faculty of Medicine, Dentistry and Health Sciences, University of Melbourne, Melbourne 3010, Australia
^7^  Molecular and Cytogenetics Laboratory, INTA University of Chile, Santiago 7830490, Chile
^8^  Psychology Service, Royal Children’s Hospital, Melbourne 3052, Australia; Lesley.Bretherton@rch.org.au
^9^  Melbourne School of Psychological Sciences, University of Melbourne, Melbourne 3052 Australia
^10^  Child Neuropsychology, Murdoch Childrens Research Institute, Royal Children’s Hospital, Melbourne 3052, Australia
^11^  Molecular Genetics Laboratory, Victorian Clinical Genetics Services and Murdoch Childrens Research Institute, Royal Children’s Hospital, Melbourne 3052, Australia; dusart.desiree@mcri.edu.au
^12^  Faculty of Medicine, University of Newcastle, Newcastle 2303, Australia; morkeu@gmail.com
^13^  Westmead Millennium Institute, University of Sydney, Westmead 2145, Australia
*  Correspondence: david.godler@mcri.edu.au; Tel.: +61-3-8341-6496; Fax: +61-3-9348-1391
**†**  These authors contributed equally to this work.

The authors would like to apologize for any inconvenience caused. The change does not affect the scientific results. The manuscript will be updated and the original will remain online on the article webpage.
